# Establishment of particulate matter-induced lung injury model in mouse

**DOI:** 10.1186/s42826-021-00097-x

**Published:** 2021-07-30

**Authors:** Se Yong Park, Kyu Sup An, Buhyun Lee, Ju-Hee Kang, Hyun Jin Jung, Min Woo Kim, Hyeon Yeol Ryu, Kyu-Suk Shim, Ki Taek Nam, Yeo Sung Yoon, Seung Hyun Oh

**Affiliations:** 1 Department of Anatomy and Cell Biology, College of Veterinary Medicine, Seoul National University, Seoul, 08826 South Korea; 2 Korea Conformity Laboratories, Gaetbeol-ro 145 beon-gil, Yeonsu-gu, Incheon, 21999 South Korea; 3 Brain Korea 21 PLUS Project for Medical Science, Severance Biomedical Science Institute, Yonsei University College of Medicine, Seoul, 03722 South Korea; 4 College of Pharmacy, Gachon University, Incheon, 21936 South Korea; 5 Univera Co., Ltd., Seoul, 04782 South Korea

**Keywords:** Air pollution, Particulate matter, Animal model, Lung injury, Asthma

## Abstract

**Background:**

Particulate matter (PM) is one of the principal causes of human respiratory disabilities resulting from air pollution. Animal models have been applied to discover preventive and therapeutic drugs for lung diseases caused by PM. However, the induced severity of lung injury in animal models using PM varies from study to study due to disparities in the preparation of PM, and the route and number of PM administrations. In this study, we established an in vivo model to evaluate PM-induced lung injury in mice.

**Results:**

PM dispersion was prepared using SRM2975. Reactive oxygen species were increased in MLE 12 cells exposed to this PM dispersion. In vivo studies were conducted in the PM single challenge model, PM multiple challenge model, and PM challenge with ovalbumin-induced asthma using the PM dispersion. No histopathological changes were observed in lung tissues after a single injection of PM, whereas mild to moderate lung inflammation was obtained in the lungs of mice exposed to PM three times. However, fibrotic changes were barely seen, even though transmission electron microscopy (TEM) studies revealed the presence of PM particles in the alveolar macrophages and alveolar capillaries. In the OVA-PM model, peribronchial inflammation and mucous hypersecretion were more severe in the OVA+PM group than the OVA group. Serum IgE levels tended to increase in OVA+PM group than in OVA group.

**Conclusions:**

In this study, we established a PM-induced lung injury model to examine the lung damage induced by PM. Based on our results, repeated exposures of PM are necessary to induce lung inflammation by PM alone. PM challenge, in the presence of underlying diseases such as asthma, can also be an appropriate model for studying the health effect of PM.

## Background

Air pollution refers to environmental pollution in which various pollutants (such as factory smoke, car exhaust, etc.) are dispersed in the atmosphere due to human activities [1]. Globally, more than 1 billion people inhale harmful indoor or outdoor air contaminated with toxic gases and particulate matters from cigarette smoke and biomass fuel [2], and 7 million people are reported to die due to such air pollution [3]. Furthermore, disabilities and deaths caused by air pollution result in significant economic losses, amounting to more than 5 trillion US dollars worldwide [4].

Among the air pollutants, fine particulate matter (PM) is a major risk factor that causes acute and/or chronic diseases in human organs, particularly the cardiovascular system [5], hepatobiliary system [6], gastrointestinal tract [7], and respiratory system [8]. PM comprises both solid and liquid particles with constantly changing size and chemical composition, and are classified depending on their diameter. PM of diameter equal to or less than 2.5 μm (PM2.5) penetrates deep into the alveoli along the airway, and is also able to traverse the alveolar wall into the blood vessels, inducing severe lung inflammation [9].

Several in vitro and in vivo studies have been conducted over the past few decades to identify the molecular mechanisms of lung diseases associated with PM exposure, and to develop preventive and therapeutic drugs. It has recently been suggested that the main event of PM-induced lung damage is intracellular ROS generation by PM, which subsequently stimulates several inflammatory signals, including the MAPK signaling pathway and NF-κB pathway, resulting in cellular injury [10–12].

As animal experiments, PM-induced lung injury mouse models have been attempted by applying various methods of PM delivery into the lungs [13] including inhalation, intratracheal instillation, endotracheal injection, and nasal inoculation. However, the severity of lung disease caused by PM differs, depending on the mode of administration. Moreover, studies have differed on the dosing of PM using the same administration method; some studies treated a single dose of PM, whereas others administered PM several times to induce lung inflammation [14].

The method of preparing PM dispersions also differed between researchers, depending on the solvents used, the number and duration of sonicating steps, and the presence of additive [15]. In addition, some studies have reported that PM alone is difficult to induce sufficient lung disease, but exposure to PM exacerbates lung injury in the presence of underlying diseases such as COPD [16] and asthma [17]. Therefore, there is a requirement to establish an in vivo model that is easy to perform, and can confirm lung damage caused by PM.

In the current study, we compared the various methods applied for preparing PM dispersions, and performed in vivo studies with differing schedules. Our experiments include both PM single and multi-challenge models, and also PM challenge in the OVA-induced airway inflammation model. This enabled us to establish a practical way to induce lung injury associated with PM in mice.

## Results

### Preparation and size analysis of PM dispersions

**Fig. 1:**
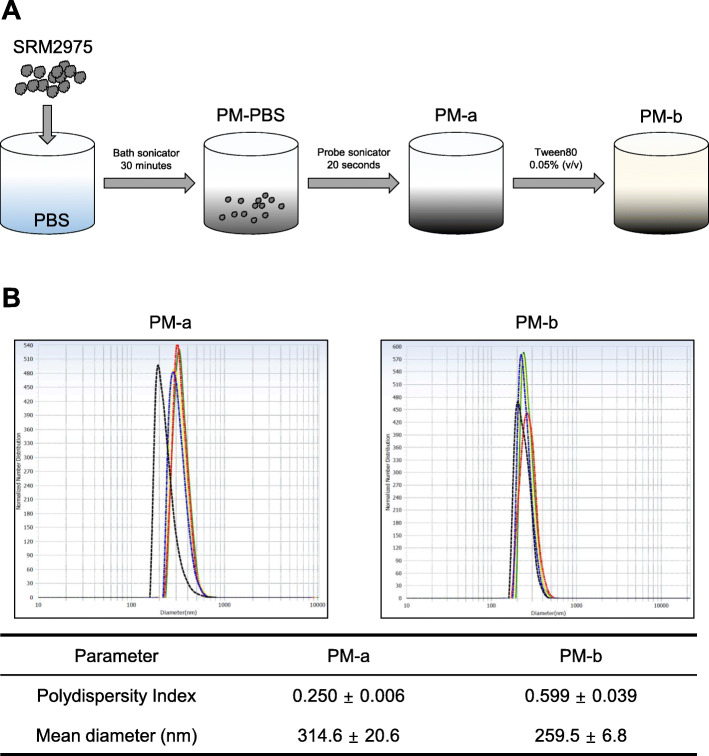
**Preparation and size analysis of particulate matter dispersions. A** Scheme of producing PM dispersions. Two PM dispersions (PM-a and PM-b) were prepared, following the procedures described. **B** Particle sizes of PM-a and PM-b were analyzed. Size distributions of each PM dispersion are shown as graphs. Polydispersity index and mean diameter of particles are described (mean ± standard error of mean)

To determine whether differences in biochemical properties are dependent on solvents, we dispersed SRM2975 in DMSO or PBS, and sonicated the suspensions using a bath sonicator (PM-DMSO and PM-PBS, respectively). MLE 12 cells were subsequently exposed to PM-DMSO or PM-PBS, and the intracellular reactive oxygen species (ROS) was measured by flow cytometry. Higher ROS levels were obtained in MLE 12 cells exposed to PM-PBS, as compared to PM-DMSO (data not shown). Size analysis for the two suspensions revealed particle sizes of PM-DMSO and PM-PBS to be 657.0 ± 43.1 nm and 413.5 ± 38.5 nm, respectively (mean ± standard error of mean (SEM)). PM-PBS was sonicated again with a probe sonicator to reduce the particle size (Fig. 1A, PM-a). Next, we investigated for differences depending on the presence of additives: PM-a was mixed with 0.05% (v/v) tween 80 (Fig. 1A, PM-b). Size analysis of PM-a and PM-b (Fig. 1B) revealed that although the polydispersity index (P.I.) of PM-b was higher than P.I. of PM-a, the mean diameter of PM-b obtained (259.5 nm) was smaller than diameter of PM-a (314.6 nm).

### Biological characteristics of PM-a and PM-b in MLE 12 cells

**Fig. 2:**
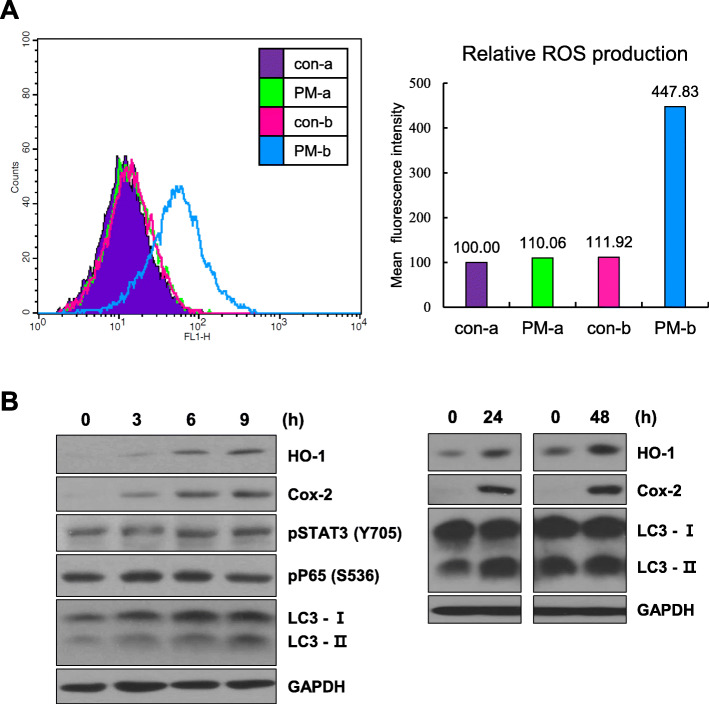
**Effects of PM-a and PM-b on the biological characteristics of MLE 12 cells. A** Intracellular reactive oxygen species are measured by flow cytometry. MLE 12 cells were treated with PM-a (100 μg/ml) or PM-b (100 μg/ml) for 30 min. Con-a and con-b indicate the control groups of PM-a and PM-b, respectively. **B** Protein expressions related to inflammatory signals and autophagy pathway were detected by Western blotting. MLE 12 cells were treated with PM-b (100 μg/ml) for the indicated hours

MLE 12 cells were exposed to PM-a or PM-b for 30 min, following which levels of intracellular ROS were measured. Compared to their respective control group, the ROS production was increased by about 10% in PM-a exposed MLE cells, whereas a 4-fold increase was determined in MLE cells treated with PM-b (Fig. 2A). This result indicates that PM-b is a more harmful stimulus than PM-a. To confirm that PM-b can induce cellular signaling related to injuries, MLE 12 cells were exposed to PM-b for indicated varying durations, and protein expression levels related to inflammatory signals or autophagy were determined by Western blotting (Fig. 2B). In short time exposures, the expression levels of HO-1 and COX-2 were time-dependently increased following PM-b exposure in MLE 12 cells, with minimal change in the pSTAT3 (Y705) and pP65 (S536) expressions. Considering the autophagy activities, the conversion of LC3-I to LC3-II showed a time-dependent increase after PM-b exposure, as compared to the control (Fig. 2B, left panel). Similar results were observed in MLE 12 cells exposed to PM-b for longer time (Fig. 2B, right panel). Based on the results of these in vitro experiments, we initiated further in vivo animal studies using PM-b.

### Pulmonary histopathology in PM single challenge model

**Fig. 3:**
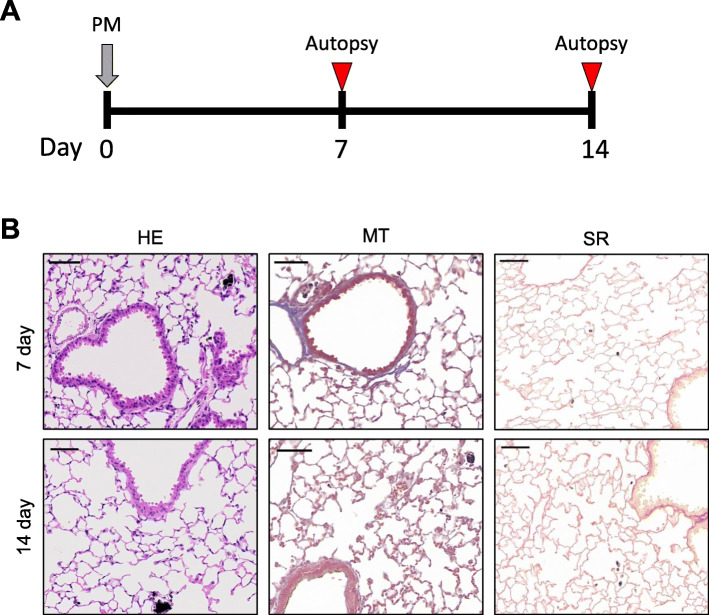
**Histopathological changes after single challenge of PM-b. A** Experimental schedule. PM-b was intratracheally injected at day 0, and mice were euthanized on days 7 (*n* = 5) and 14 (*n* = 5). **B** Representative images of the lung of mice on days 7 and 14. Pulmonary histopathological changes after single challenge of PM-b were evaluated by three different types of staining (HE: Hematoxylin-Eosin staining; MT: Masson’s Trichrome staining; SR: Sirius Red staining). Scale bar: 100 μm

To explore the effect of duration from single dose of PM to autopsy, intratracheal instillation of PM-b was performed for ICR mice at day 0. Mice were divided into two groups: mice euthanized at day 7 (*n* = 5) or day 14 (*n* = 5) (Fig. 3A). On the day of autopsy, the lungs were harvested and histologically evaluated by subjecting the lung tissue to HE staining. PM-b alone and alveolar macrophages containing PM-b were detected, but there were no observable features related to lung injury such as thickened alveolar walls and infiltration of inflammatory cells. Similarly, MT and SR staining revealed no significant fibrotic changes in the lung (Fig. 3B, above). And also, there were no obvious histological differences between day 7 and day 14 (Fig. 3B, below).

### Pulmonary histopathology in the PM multiple challenges model

**Fig. 4:**
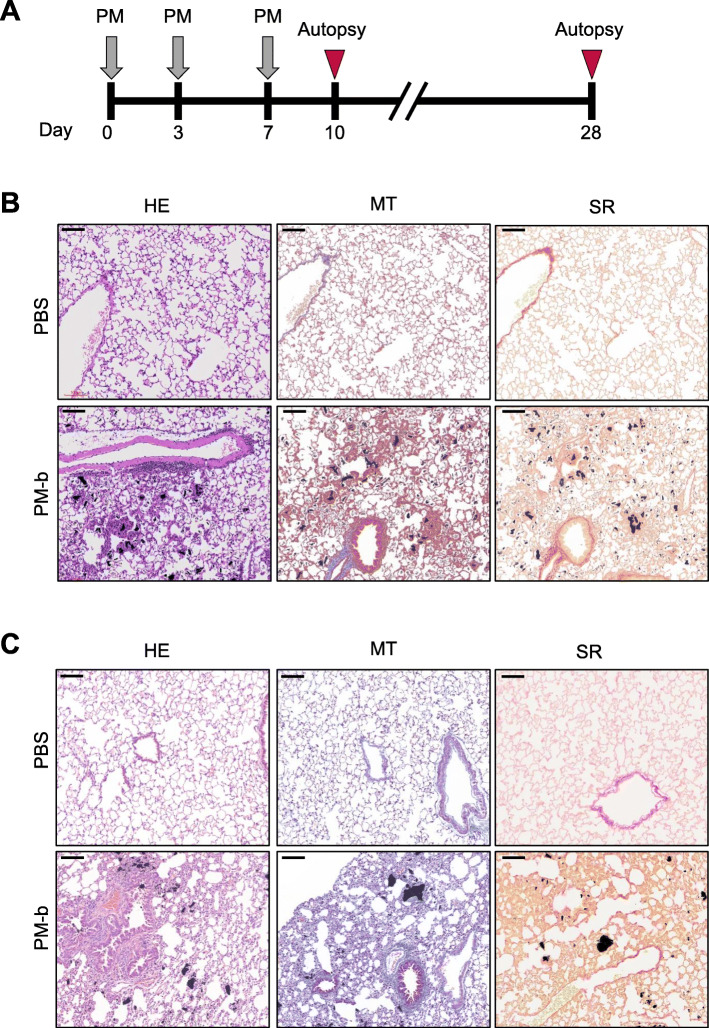
**Histopathological changes after multiple challenges of PM-b. A** Experimental schedule. PM-b or PBS were intratracheally injected on days 0, 3, and 7. Mice were euthanized on day 10 (PBS injected mice: *n* = 5; PM-b injected mice: *n* = 10) or day 28 (PBS injected mice: *n* = 5; PM-b injected mice: *n* = 8). **B, C** Representative images of the lung of mice on days 10 (**B**) and 28 (**C**). Pulmonary histopathological changes were evaluated by three different types of stainings (HE: Hematoxylin-Eosin staining; MT: Masson’s Trichrome staining; SR: Sirius Red staining). Scale bar: 100 μm

In order to verify that multiple administrations of PM-b induces pulmonary inflammation, ICR mice were intratracheally injected with PM-b three times, and autopsy was performed 3 days or 3 weeks after the last injection (Fig. 4A). HE staining of the lungs harvested at day 10 revealed an increased number of PM-b depositions compared to lungs examined in the single challenge model. Moreover, mild inflammation was also observed, as compared to the PBS control group. Nevertheless, significant histopathological changes related to lung fibrosis were not detected on MT and SR staining (Fig. 4B). On day 28 after the last injection, HE staining revealed moderate inflammation and an increased number of PM-b depositions in the lungs of PM-b exposed mice, but there was no evidence of pulmonary fibrosis on MT and SR staining (Fig. 4C).

### Transmission Electron Microscopy (TEM) images of the lung treated with PM-b

**Fig. 5:**
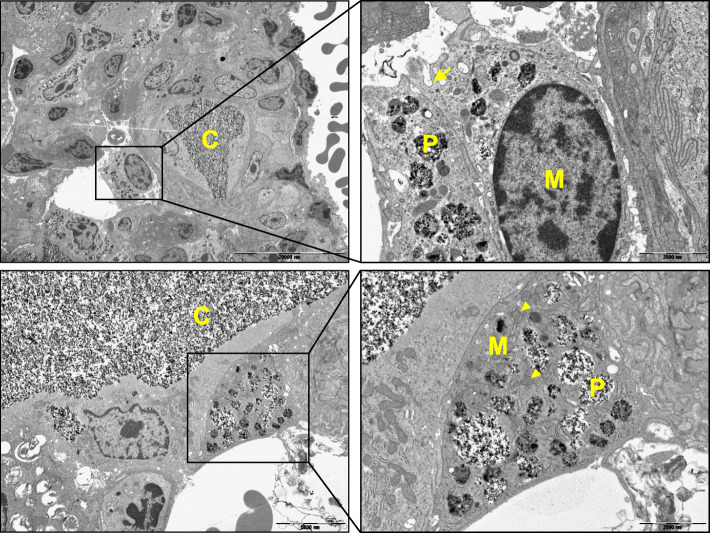
**Transmission electron microscopy images of lungs treated with PM-b**. At day 28, some parts of the lung tissues were fixed and subjected to TEM. PM-b particles were observed in alveolar macrophages and alveolar capillaries. P: vacuoles containing PM-b particles; M: alveolar macrophage; C: alveolar capillary; Arrow: pinocytotic vesicle; Arrowhead: mitochondria. Scale bar: 20,000 nm and 2000 nm in low and high magnification images, respectively

Transmission electron microscopic images were taken to examine for phagocytosis of PM-b by alveolar macrophages. After three administrations of PM-b (Fig. 4A, day 28), several vacuoles containing PM-b were observed in the alveolar macrophages, and PM-b depositions were also observed in the alveolar capillaries (Fig. 5).

### Histopathological changes and serum IgE ELISA in the OVA-PM model

**Fig. 6:**
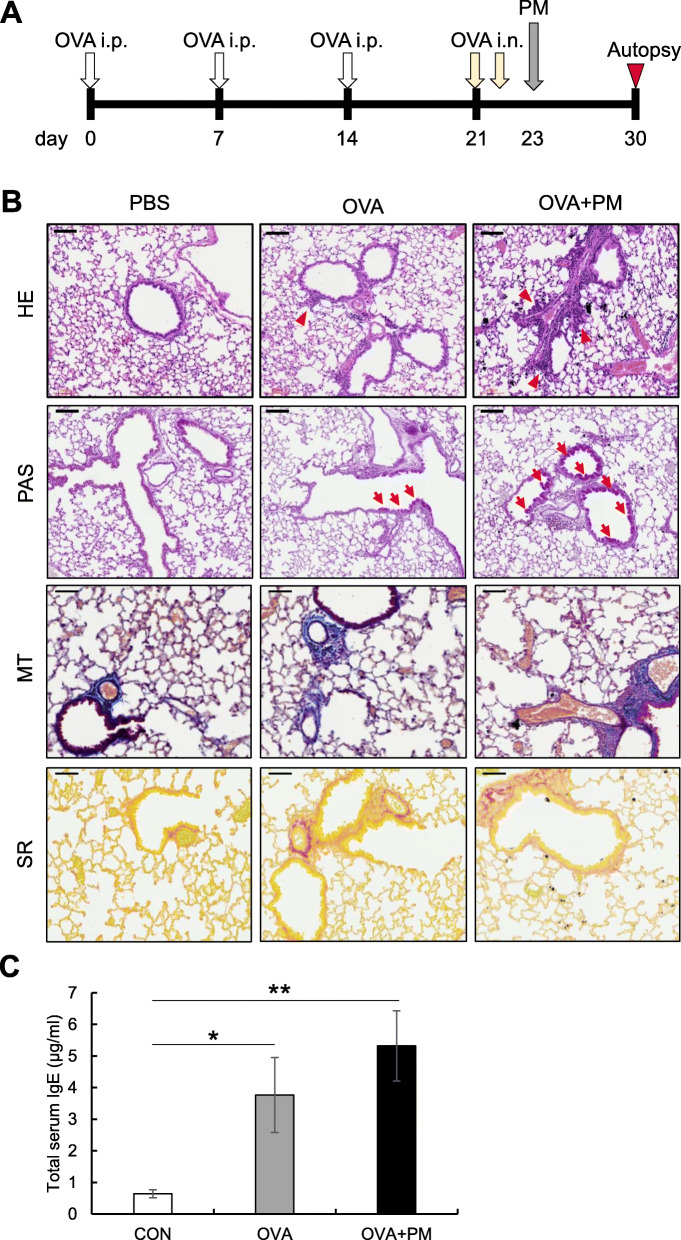
**Histopathological changes in the lungs of mice obtained from the OVA-PM model. A** Experimental schedule. OVA or PBS were intraperitoneally injected on days 0, 7, and 14. Thereafter, ovalbumin or PBS were intranasally inoculated on days 21 and 22, followed by intratracheally injection of PM-b or PBS on day 23. All mice were euthanized on day 30 (CON group: *n* = 5; OVA group: *n* = 6; OVA+PM group: *n* = 5). **B** Representative images of the lungs of mice at the day of autopsy. Infiltration of inflammatory cells were observed in the OVA group and OVA+PM group (Red arrow head on the HE staining). The number of infiltrated cells were increased in the OVA+PM group, as compared to the OVA group. The number of PAS positive cells were greater in lungs obtained from the OVA+PM group than the OVA group (Red arrow on the PAS staining). Scale bar: 100 μm. **C** Serum total IgE of all mice was measured by ELISA and compared between groups. The bar graphs are presented as mean ± standard error of mean. One-way ANOVA test was used for statistical analysis. * *p* < 0.05, ** *p* < 0.01

To determine the effect of PM-b exposure on mice with lung inflammation induced by OVA, we conducted the OVA-PM model according to the following experimental scheme (Fig. 6A). To induce pulmonary inflammation using OVA, mice were sensitized with intraperitoneal injections of OVA complexed with Al(OH)_3_, and challenged with intranasal inoculation of 2% OVA solution. In the lung tissues obtained from the OVA group, HE staining revealed infiltration of inflammatory cells in the peribronchial regions, and also an increased number of PAS positive cells, as compared to that of the control group. Moreover, the number of infiltrated cells and PAS positive cells were greater in the OVA+PM group, as compared to the OVA group (Fig. 6B). To compare the allergic reaction among groups, serum IgE levels were determined by ELISA. Compared to the control groups, serum IgE levels were elevated in both the OVA and OVA+PM groups. Although not significant, the level tended to increase in response to PM-b exposure (Fig. 6C).

## Discussion

The introduction of various aerosol pollutants into the air results in environmental air pollution, and 7 million people die annually due to inhalation of toxic gases and PM produced by combustion of cigarettes and biomass fuels. In addition, economic losses amounting to trillions of US dollars have been incurred in relation to the deaths caused by air pollution. PM, one of the main risk factors of air pollution, enters the lower respiratory tract and results in lung inflammation. Especially, PM2.5 is able to penetrate into the blood vessels and cause a variety of diseases across organs in humans.

For the past few decades, in vitro and in vivo studies have been conducted to identify the molecular biological mechanisms of diseases caused by PM, and to establish preventive measures or develop treatments. The PM-induced lung disease model in mice has been widely applied in these researches, but each study has a different degree of inflammation or fibrosis with the same administration method and dosing schedule. In the current study, animal experiments were performed using several methods, with the intention to establish a practical protocol to reproduce PM-induced lung damage in humans.

Prior to animal experiments, we established a procedure for making PM dispersion, since varied methods had been used by different researchers. Recent studies have suggested that PM induces cellular damage through ROS in a variety of cells, such as pneumocytes [12], macrophages [18], and endothelial cells [19]. We therefore focused on ROS generation induced by the PM dispersion prepared in our laboratory.

Considering that the compositional variation of PM depends on the origin of the collected PM, we conducted the in vitro and in vivo experiments using SRM2975 as a reference material. SRM2975 was first dispersed in either DMSO or PBS to explore the variations produced by different solvents. Intracellular ROS level was higher in MLE 12 cells treated with PM-PBS than with PM-DMSO exposure. Moreover, the mean particle size of PM-PBS was also smaller than that of PM-DMSO (413.5 nm and 657.0 nm, respectively). These sizes were small enough to meet the PM2.5 criteria, but larger than the reported size in other studies using SRM2975 [20–22]. We therefore prepared another PM dispersion containing smaller size of particles by sonicating the PM-PBS (Fig. 1A, PM-a). One study suggested that PM dispersions with stabilizers such as human serum albumin and tween 80 are stable without forming coarse agglomerates [15]. Following this concept, PM-b was prepared by mixing PM-a with tween 80 (Fig. 1A, PM-b).

Size analysis of PM-a and PM-b revealed that PM-b had a smaller mean diameter than PM-a (Fig. 1B). Higher ROS was produced in MLE 12 cells treated with PM-b than with PM-a (Fig. 2A), thereby confirming that smaller particles cause a more severe lung disease [23]. Furthermore, Western blotting analysis confirmed an increase in protein expression levels of inflammatory signals and autophagic activities after PM-b exposure in MLE 12 cells (Fig. 2B). Based on the results of induced intracellular ROS, increased inflammatory signals, and increased autophagy activities, subsequent animal experiments were conducted using PM-b.

Considering the exposure route of PM in humans, the inhalation method may produce similar results with the consequences observed in humans exposed to PM [24]. However, since it is challenging to perform the inhalation method due to the need for dedicated equipment (such as aerosol generator and ventilating system), the current study applied the intratracheal instillation method to deliver PM into the lung.

The single challenge model was performed in mice to induce lung inflammation using a single dose of PM-b. Mice exposed to PM-b were sacrificed at 7 days or 14 days after PM-b exposure, to evaluate differences in lung injury depending on the retention period of PM-b in the body (Fig. 3A). At 7 days after i.t. instillation of PM-b, the PM-b itself and alveolar macrophages containing PM-b were observed in the lungs. However, neither lung inflammation or lung fibrosis were induced. Similar results were observed in the lungs of mice sacrificed at day 14 (Fig. 3B).

It was therefore confirmed that a single intratracheal injection of PM-b is insufficient to induce lung damage. Studies conducting animal experiments that inject PM several times to induce lung damage have also been reported [25–27]; hence, we also applied the multiple challenge model (Fig. 4A). After three doses of intratracheal instillation of PM-b, TEM images revealed the presence of PM-b particles in the vacuoles of alveolar macrophages, and also in blood vessels (Fig. 5). Histological evaluation determined greater PM-b accumulation in lung tissues after three doses of intratracheal instillation of PM-b, and more macrophages with PM-b containing vacuoles were observed. Although there was evidence of mild to moderate lung inflammation, such as thickening of alveolar walls and infiltration of inflammatory cells, there were no findings of pulmonary fibrosis (Fig. 4B, C).

In the previous two animal experiments (single challenge model and multiple challenges model), only PM-b alone was unable to reproduce lung damage accompanied by lung fibrosis. In addition, in studies with repeated intratracheal injections, the results tended to depend significantly on the experimenter’s proficiency in surgical processes. It has recently been reported that PM contributes to increasing the lung damage in patients with underlying diseases such as COPD and asthma [28]. We therefore performed the OVA-PM model to confirm lung damage caused by PM-b, and simultaneously reducing the difference between experimenters by minimizing invasive processes (Fig. 6A).

Notable histological features in asthmatic patients include mucous hypersecretion, fibrosis in subepithelial region, inflammatory cell infiltration, and hypertrophy of bronchial smooth muscle [29]. These histopathological changes in asthma are mainly mediated by IgE, since asthma is a type I hypersensitivity reaction [30]. In the animal experiment applying the OVA-PM model, increased PAS positive cells and peribronchial infiltration of inflammatory cells were observed in the OVA-induced asthma models (Fig. 6B, OVA). Also, significantly higher levels of serum total IgE were detected, as compared to the control group (Fig. 6C, OVA). Furthermore, not only peribronchial inflammation was more severe, but the number of PAS positive cells was increased in mice exposed to PM-b after inducing asthma, than mice of the OVA group. The total IgE level in serum also tended to increase in the combined treatment as compared to the only OVA group (Fig. 6B, C). Deposition of collagen was slightly increased after OVA treatment, but there was no significant differences between OVA and OVA+PM group (Fig. 6B).

Previous studies have reported that the profile of inflammatory cells in bronchoalveolar lavage fluid (BALF) is altered after PM exposure [26], and inflammatory cytokines such as tumor necrosis factor alpha (TNF-α), interleukin 1 beta (IL-1β), and interleukin 6 (IL-6) are increased in BALF of mice treated with PM [31–33]. However, the above findings from BALF analysis are more meaningful when histopathological changes occur simultaneously; hence, in the present study, we focused on inflammatory and fibrous lesions caused by PM.

As an in vivo model to study the mechanism of PM-induced lung injury, it was observed that it is difficult to reproduce lung diseases by intratracheal instillation of PM alone. Therefore, we recommend the OVA-PM model established in this work as an appropriate animal model, for studying the adverse effects of PM.

## Conclusions

In conclusion, this study establishes a method to prepare PM dispersion and a PM-induced lung injury model for in vivo studies, to verify the effects of PM exposure on lungs in mice. PM-b was generated by sonicating SRM2975, followed by addition of tween 80 as an adjuvant. This preparation produces higher intracellular ROS in MLE 12 cells than other PM dispersions. Based on the results from animal experiments, it was found that a single intratracheal instillation of PM-b is insufficient to induce lung inflammation in mice. Also, no significant pulmonary fibrosis was observed after multiple PM challenges, even up to three doses. However, PM-b aggravated the OVA-induced asthma in the OVA-PM model. Therefore, we suggest that intratracheal instillation of PM in the presence of underlying diseases is potentially a suitable animal model for studying molecular biological mechanisms of PM-induced lung injury, and for researching developing therapeutics against such injuries.

## Methods

### Reagents

Diesel particulate matter SRM 2975 was purchased from the National Institute of Standards and Technology (NIST, Gaithersburg, USA). The MLE 12 murine type II pneumocyte cell line was obtained from ATCC (Manassas, VA, USA). The fluorogenic dye 2 ´,7 ´-dichlorodihydrofluorescein diacetate (DCFH-DA), ovalbumin (OVA) and aluminum hydroxide (Al(OH)_3_) were purchased from Sigma-Aldrich (St Louis, MO, USA). Primary antibodies were purchased from several vendors: COX-2 (Santa Cruz Biotechnology, Santa Cruz, CA, USA), HO-1 (R&D systems, Minneapolis, MN, USA), pSTAT3, pP65, and LC3A/B (Cell Signaling Technology, Danvers, MA, USA), and GAPDH (Merck Millipore, Darmstadt, Germany). Glutaraldehyde, paraformaldehyde, and absolute ethanol were obtained from Merck Millipore, osmium tetroxide (OsO_4_) was obtained from Polysciences (Warrington, PA, USA), and 99% pure propylene oxide was obtained from Acros Organics (99% pure, Morris Plains, NJ). ELISA kits for mouse serum IgE were purchased from eBioscience (San Diego, CA, USA).

### Preparation and size analysis of PM dispersions

Prior to making PM-a and PM-b, SRM2975 was dispersed in phosphate buffered saline (PBS) at a final concentration of 2.5 mg/ml (PM-PBS). For making PM-a, the PM-PBS was sonicated in a bath sonicator for 30 min and probe sonicator for 20 s. PM-b was made by mixing PM-a with tween 80 at 0.05% (v/v). All dispersions were stored at − 20 °C, until further use. Size analysis of PM dispersions was conducted by a zeta potential and particle size analyzer (ELSZ-1000, Otsuka Electronics CO, Osaka, Japan), as previously described [34]. Briefly, each PM dispersion was diluted 1:100 in PBS to a total volume of 1 ml. The mean diameter, polydispersity index (P.I.), and number distribution were subsequently measured for the particles contained in each dispersion, and this procedure was repeated five times for each dispersion.

### Cell cultures

MLE 12 cells were cultured in Dulbecco’s Modified Eagle Medium (DMEM) supplemented with 2% (v/v) fetal bovine serum (FBS) and penicillin/streptomycin (Welgene, Daegu, Korea). For all in vitro studies described below, MLE 12 cells were seeded in 12-well and 6-well cell culture plates at a cell seeding density of 5 × 10^5^ cells per well and 1 × 10^6^ cells per well, respectively. Next day, the cells were washed with PBS and exposed to 100 μg/ml PM for the indicated hours. The treated MLE 12 cells were then subjected to intracellular reactive oxygen species (ROS) measurement or Western blotting.

### Intracellular ROS measurement

Intracellular ROS was measured using DCFH-DA, as previously described [35]. Briefly, MLE 12 cells were treated with PM for 30 min, followed by staining with DCFH-DA (2.5 μM) for 20 min. After staining, the cells were washed with PBS and harvested. The harvested cells were centrifuged at 1400 rpm for 3 min, and the cell pellet was washed with PBS and re-centrifuged. The washed cell pellet was resuspended in PBS and subjected to flow cytometry for measuring intracellular ROS.

### Western blotting

MLE 12 cells were exposed to PM-b for 3, 6, 9, 24, or 48 h, and the total protein obtained was subjected to Western blotting. Briefly, the PM treated cells were lysed with radioimmunoprecipitation assay (RIPA) buffer and the total protein was extracted. Quantification of the extracted protein was performed, and identical amounts of protein were separated by SDS-PAGE. The proteins on SDS-PAGE gel were transferred to polyvinylidene fluoride membranes, followed by blocking the membranes with 5% (w/v in TBS-T) skim milk. After washing, blocked membranes were incubated with the respective primary antibodies, overnight at 4 °C. Next day, the primary antibody conjugated membranes were incubated with the secondary antibody for 1 h. Detection of the immunoreactive bands was performed using Absignal (Abclon, Seoul, Korea).

### In vivo experiments

For animal experiments, ICR and BALB/c male mice were purchased from Orient Bio (Seungnam, Korea). All animal experiments were conducted at Gachon University, in accordance with the procedures approved by the Institutional Animal Care and Usage Committee (IACUC) (GIACUC-R2020020–1). For this study, we conducted three animal experiments: (1) PM single challenge model, (2) PM multiple challenges model, and (3) PM challenge with ovalbumin (OVA)-induced asthma (OVA-PM) model.

1. PM single challenge model

Seven-week-old male ICR mice were acclimated for 1 week, and all mice were exposed to PM-b at 250 μg/mouse through intratracheal (i.t.) injection, as previously described (day 0) [36]. Mice were euthanized at day 7 (*n* = 5) and day 14 (*n* = 5).

(2) PM multiple challenges model

Seven-week-old male ICR mice were randomly divided into two groups after 1 week acclimation; each group was exposed to either PBS or PM-b (250 μg/mouse), through i.t. injection on days 0, 3, and 7. Mice were euthanized on day 10 (PBS injected mice: *n* = 5; PM-b injected mice: *n* = 10) or day 28 (PBS injected mice: *n* = 5; PM-b injected mice: *n* = 8).

(3) PM challenge with ovalbumin (OVA)-induced airway inflammation (OVA-PM) model.

Seven-week-old male BALB/c mice (*n* = 16) were purchased from Orient Bio (Seongnam, Korea). Mice were randomly divided into three groups after acclimation for 1 week (CON group: *n* = 5; OVA group: *n* = 6; and OVA+PM group: *n* = 5). Mice of OVA and OVA+PM groups were sensitized with 10 μg OVA emulsified in 1.5 mg Al(OH)_3_ in 200 μl of PBS, through intraperitoneal injection, on days 0, 7, and 14. Mice of the OVA and OVA+PM groups were anesthetized by intraperitoneal injection of Avertin (20 mg/kg), and challenged with 2% (w/v) OVA solution, 25 μl per nostril per mouse on days 21 and 22. On day 23, mice of the OVA+PM group were exposed to PM-b at 250 μg/mouse by administering an i.t. injection, while mice of the OVA group were exposed to only PBS. Mice of the CON group received only PBS on the same day through the same route. All mice were euthanized at day 30.

On the day of autopsy in all three experiments, blood was collected through cardiac puncture, and the serum was separated from each blood sample. The lungs were perfused with sterile PBS, and subsequently harvested for histological examination.

### Histological examination

To investigate histopathological changes, the lungs were harvested on the day of autopsy and fixed in 10% neutral buffered formalin, followed by embedding in paraffin blocks. The paraffin blocks were then sectioned at 5 μm-thick slices. All lung sections were subjected to HE staining to evaluate the severity of alveolar wall thickness and inflammatory cells infiltration. MT and SR staining were also performed to evaluate fibrotic changes in response to PM exposure. PAS staining was conducted to compare the severity of mucous hypersecretion in lungs of mice of the OVA-PM model. Each stained section was observed under light microscope.

### Transmission Electron Microscopy (TEM)

Specimens for transmission electron microscopy were fixed for 12 h in 2% glutaraldehyde-2% paraformaldehyde prepared in 0.1 M phosphate buffer (pH 7.4), subsequently washed in 0.1 M phosphate buffer, post-fixed with 1% OsO4 in 0.1 M phosphate buffer for 2 h, and dehydrated with an ascending ethanol series (50, 60, 70, 80, 90, 95, 100, 100%) for 10 min each, followed by infiltration with propylene oxide for 10 min. Specimens were embedded with a Poly/Bed 812 kit (Polysciences), and polymerized in an electron microscope oven (TD-700, DOSAKA, Japan) at 65 °C for 12 h. The block subsequently cut into 200 nm semi-thin section using a diamond knife in the ultramicrotome. Then, the sections were stained with toluidine blue and observed under an optical microscope. The region of interest was then cut into 80 nm thin sections using the ultramicrotome. The sections were placed on copper grids and double stained with 3% uranyl acetate and 3% lead citrate for 30 min and 7 min, respectively. The stained sections were then imaged with a transmission electron microscopy (JEM-1011, JEOL, Tokyo, Japan) equipped with a Megaview III CCD camera (Soft imaging system-Germany), at an acceleration voltage of 80 kV.

### Serum IgE enzyme-linked immunosorbent assay (ELISA)

Total serum IgE levels were detected using the commercially available ELISA kit, following the manufacturer’s recommendation. The serum samples were diluted (1:25) and subjected to IgE ELISA.

### Statistical analysis

To compare serum IgE levels between groups, One-way ANOVA test was used for statistical analysis. Experimental data are expressed as mean ± standard error of mean.

## Data Availability

Not applicable.

## Abbreviations

BALF: Bronchoalveolar lavage fluid
COPD: Chronic obstructive pulmonary disease
DCFH-DA: 2 ´,7 ´-dichlorodihydrofluorescein diacetate
DMEM: Dulbecco’s modified eagle medium
DMSO: Dimethyl sulfoxide
ELISA: Enzyme-Linked Immunosorbent Assay
HE: Hematoxylin & Eosin
HRP: Horseradish peroxidase
IgE: Immunoglobulin E
IL-1β: Interleukin 1 beta
IL-6: Interleukin 6
MT: Masson’s trichrome
OVA: Ovalbumin
PAS: Periodic acid Schiff
PBS: Phosphate buffered saline
P.I.: Polydispersity index
PM: particulate matter
PM2.5: PM of which diameter is even or less than 2.5 μm
PVDF: Polyvinylidene fluoride
RIPA buffer: Radioimmunoprecipitation assay buffer
ROS: Reactive oxygen species
SR: Sirius red
TEM: Transmission electron microscopy
TNF-α: Tumor necrosis factor alpha
